# In-Vivo fluorescent nanosensor implants based on hydrogel-encapsulation: investigating the inflammation and the foreign-body response

**DOI:** 10.1186/s12951-023-01873-8

**Published:** 2023-04-24

**Authors:** Michael A. Lee, Xiaojia Jin, Sureshkumar Muthupalani, Naveed A. Bakh, Xun Gong, Michael S. Strano

**Affiliations:** 1 Department of Chemical Engineering, Massachusetts Institute of Technology, Cambridge, MA 02139 USA; 2 Division of Comparative Medicine, Massachusetts Institute of Technology, Cambridge, MA 02139 USA

**Keywords:** Nanosensor, Carbon nanotube, Inflammation, Hydrogel, Implants

## Abstract

Nanotechnology-enabled sensors or nanosensors are emerging as promising new tools for various *in-vivo* life science applications such as biosensing, components of delivery systems, and probes for spatial bioimaging. However, as with a wide range of synthetic biomaterials, tissue responses have been observed depending on cell types and various nanocomponent properties. The tissue response is critical for determining the acute and long term health of the organism and the functional lifetime of the material *in-vivo*. While nanomaterial properties can contribute significantly to the tissue response, it may be possible to circumvent adverse reactions by formulation of the encapsulation vehicle. In this study, five formulations of poly (ethylene glycol) diacrylate (PEGDA) hydrogel-encapsulated fluorescent nanosensors were implanted into SKH-1E mice, and the inflammatory responses were tracked in order to determine the favorable design rules for hydrogel encapsulation and minimization of such responses. Hydrogels with higher crosslinking density were found to allow faster resolution of acute inflammation. Five different immunocompromised mice lines were utilized for comparison across different inflammatory cell populations and responses. Degradation products of the gels were also characterized. Finally, the importance of the tissue response in determining functional lifetime was demonstrated by measuring the time-dependent nanosensor deactivation following implantation into animal models.

**Supplementary Information:**

The online version contains supplementary material available at 10.1186/s12951-023-01873-8.

## Introduction

Nanosensors have increasingly been utilized for *in vivo* biological applications, including studies of intra- and inter-cellular signaling pathways, environmental sensing, as well as continuous monitoring of spatio-temporal dynamics of particular analytes [1]. Recent advances [2–6] in technology have produced nanosensors that have the potential to revolutionize medical technology by providing real-time quantitative measurements of the human biochemical signaling network, enabling new, previously difficult modalities of diagnostics and treatment. Depending on the application, nanosensors have been employed as injectable liquids or encapsulated in a hydrogel matrix as solid implants [1, 7–10]. The latter [9, 10] has been shown to render materials more biocompatible and is a widely employed strategy to interface nanomaterials with living systems. While various studies have investigated the cellular toxicity of nanomateirals and their decomposition fragments [11–15], few have examined the effect of hydrogel formulation on *in vivo* nanomaterial toxicity. In this study, we study the tissue responses of five nanoparticle hydrogel formulations implanted subcutaneously in SKH1-E mice, using near-infrared (nIR) fluorescent single walled carbon nanotube (SWNT) as a model nanosensor to address the link between hydrogel formulation and tissue responses.

As an emerging nanomaterial for biological applications, a central question regarding SWNT has been the types of chemical functionalization or matrices that enable biocompatibility. It is important to note, however, that SWNT comprises a family of materials characterized by different diameters, lengths, and methods of synthesis, all of which have important effects on biocompatibility [2, 16]. Numerous studies have investigated the biocompatibility of SWNTs *in vitro*. It has been observed that the synthesis method plays a crucial role in toxicity due to the presence of impurities such as metal catalysts. For example, the high-pressure carbon monoxide (HiPCO) process utilizes iron (Fe) catalysts [17] to produce SWNTs, while the cobalt-molybdenum catalyst (CoMoCAT) process uses CoMo catalysts [18]. Residual catalysts appear to drastically alter SWNT cytotoxicity [11]. Furthermore, SWNT length has been shown to alter its biodistribution properties, with shorter, individualized SWNT (< 300 nm) able to be cleared by the kidneys and larger SWNT aggregates inducing granuloma formation and phagocytosis [19]. Furthermore, the SWNT corona, or the polymeric layer wrapping around the SWNT, has been reported to alter SWNT cytotoxicity. Dong et al. reported that SDS and SDBS-wrapped SWNT reduced 1321N1 human astrocytoma cell viability, while sodium cholate and DNA-wrapped SWNT did not affect cell viability or proliferation [13]. Given the heterogeneity of cellular responses and SWNT functionalization methods, the toxicity of each unique SWNT construct may have to be considered individually if used in direct contact with tissues. Lastly, it is noteworthy that these effects are independent of the use of an encapsulation matrix such as a hydrogel, and therefore require consideration.

While a useful preliminary step [20], *in vitro* test results might not be representative of the real *in vivo* tissue responses to a compound because of heterogeneous cell populations *in vivo*, different effective doses and exposure times due to *in vivo* systems being open as opposed to closed as in cell culture [21], and chemical transformation of the material upon introduction into the body [22]. Thus, direct testing in the *in vivo* environment [23] is critical as it represents the real route, dose, and location of administration.

In recent years, SWNTs have become widely used *in vivo*. Iverson et al. delivered DNA-wrapped SWNT via tail vein injection into mouse livers to detect the onset of inflammatory events [9]. The authors also implanted SWNT encapsulated in alginate hydrogels subcutaneously, where they remained for 400 days. Histological analysis showed minimal inflammation at the implantation site. Williams et al. and Harvey et al. encapsulated DNA-wrapped SWNT into dialysis bags and implanted them into mice intraperitoneally [8, 24]. Jena et al. delivered DNA-wrapped SWNT into the liver to detect endolysosomal lipid flux in the liver [22]. Our group has previously encapsulated DNA-wrapped SWNT into poly(ethylene glycol) diacrylate (PEGDA) hydrogels and implanted them into various marine animals to study stress levels and hormonal signaling [10]. High-resolution ultrasound found that changes in tissue architecture were negligible in the catshark and eel, while histological analysis found evidence of a foreign body reaction in the turtle.

These studies together show the potential of SWNT to be used in various *in vivo* applications. However, even when tissue responses were reported, these studies did not explore the modification of tissue responses based on changes in SWNT formulation. Given the heterogeneity of toxic responses observed based on SWNT factors and biomaterials in general [16, 25], there is potential to maintain SWNT function while minimizing adverse tissue responses through the careful formulation of the delivery vehicle.

In this study, five hydrogel formulations were implanted into SKH1-E mice. Formulation parameters included SWNT concentration, hydrogel cross-linking density, and SWNT wrapping. Tissue samples around the implant were collected at various times and characterized in terms of the inflammatory infiltrate and thickness of fibrous capsule formation. From these results, we produce candidate design rules to formulate SWNT systems for minimal tissue responses. Furthermore, the effect of tissue responses on SWNT sensor functionality was characterized.

## Methods and materials

### Materials

Raw single walled carbon nanotubes (SWNT) were purchased from NanoIntegris (Batch HR27-104). Poly (ethylene glycol) diacrylate (PEGDA) (M_n_ = 8000) was purchased from Alfa Aesar, while PEGDA M_n_ = 1000 was purchased from Sigma Millipore. Unless otherwise noted, the remaining reagents were purchased from Sigma Millipore.

### Synthesis and Characterization of Hydrogels

SWNT were encapsulated in PEGDA hydrogels matrix using a modified version of a previously reported protocol [9]. PEGDA (100 mg/L), SWNT (25 mg/L) and 2-hydroxy-4’-(2-hydroxyethoxy)-2methylprriophenone (0.175 mg/mL for PEGDA 8000 or 1.75 mg/mL for PEGDA 1000) were mixed in 1x phosphate buffered saline (PBS) and placed into a glass mold. The mixture was held under a nitrogen atmosphere for 15 min and subsequently cross-linked under 365 nm UV light (UVP Blak-Ray XX-15BLB, 15 W) for 60 min. The solid hydrogels were incubated in 1x PBS with regular buffer replacements for at least 5 days to remove unencapsulated SWNT and unreacted reagents. Formulations are listed in Table 1.

**Table 1:** Hydrogel Formulations

Gel	SWNT (mg/L)	PEGDA (g/mol)
1	SM8-3 (25 mg/L)	8000
2	(AAAT)_7_ (25 mg/L)	8000
3	SM8-3 (25 mg/L)	1000
4	Blank	8000
5	Blank	1000

Hydrogel pore sizes were estimated using a swelling protocol previously reported. The mass of the swollen hydrogel and a dehydrated hydrogel was measured. Equations (1)-(3) were used to estimate the pore size [26]:

1 $$Q=\frac{{{m_{{\text{swollen}}}}}}{{{m_{{\text{dry}}}}}}={\alpha ^{ - 1}}$$

2 $${\bar {M}_c}^{{ - 1}}=\frac{2}{{{{\bar {M}}_n}}} - \frac{{\left( {\bar {v}/V} \right)\left[ {{\text{ln}}\left( {1 - \alpha } \right)+\alpha +\chi {\alpha ^2}} \right]}}{{{\alpha ^{1/3}} - (2/\theta )\alpha }}$$

3 $$\xi ={\alpha ^{ - 1/3}}{\left( {\frac{{2{C_\infty }{l^2}{{\bar {M}}_c}}}{{{M_0}}}} \right)^{1/2}}$$

where *Q* is the hydrogel mass swelling ratio, *m* is mass, where ${{\bar M}_C}$ is the molecular weight between cross links, ${{\bar M}_n}$ is the molecular weight of the precursor polymers, $\bar v$ is the specific volume of the polymer (= 0.903 mL/g), *V* is water’s specific volume (= 18.01 mL/mol), *χ* is the Flory-Huggins parameter (= 0.3765)[27], *θ* is the functionality of PEGDA (= 4), ξ is the average pore size, C_∞_ is the Flory characteristic ratio (= 6.9), *l* is the length of carbon-carbon bonds (= 0.154 nm), and *M*_*0*_ is the molar mass of the monomeric unit (= 44.05 g/mol).

Fluorescence imaging on hydrogels was conducted with a 2D InGaAs camera (Princeton Instruments) coupled to a Nikon AF Micro-Nikkor 60 mm 4/2.8D lens. The hydrogels were excited by a 785 nm Invictus laser (Kaiser). The optical window from 1075 to 1200 nm was monitored using a 1075 nm long-pass filter and a 1200 nm short-pass filter (Edmund Optics). Sensor responses to progesterone were tested by placing hydrogels in 6-well plates and exposed to varying concentrations of progesterone.

### Mouse surgeries and tissue collection

All animal procedures were reviewed and approved by the Committee on Animal Care at MIT. Hydrogels were autoclaved at 121 ℃ for 30 min before implantation. Female 7 week old mice (Charles River Laboratory and Jackson Labs) were anesthetized using 2% isoflurane gas. Once unresponsive, the implantation sites were sterilized using alternating washes with iopovidone and 70% ethanol repeated thrice. Hydrogels were implanted subcutaneously in the dorsal side of the animal. Two hydrogels would be implanted at a time. Implantation sites were sutured using vicryl stitches (Ethicon). SKH1-E mice line were used as immunocompetent control, while four other immunocompromised mouse line (NOD.Cg-Prkdcscid Il2rgtm1Wjl/SzJ, CB17.Cg-Prkdc^scid^Lyst^bg−J/^Crl, CB17/lcr-Prkdc^scid^/lcrlcoCrl, CAnN.Cg-Foxn1^nu^/Crl) were used to compare the effect of different inflammatory cell populations and responses.

At the appropriate time point (1, 7, 14, or 28 days), mice were euthanized by CO_2_ asphyxiation. Tissue samples were collected around the hydrogel implantation sites and a control surgical wound site without a hydrogel to give a surgical baseline. Tissues were fixed in 10% formalin and subjected to H&E staining for histological analysis.

### Analysis of degradation products

Hydrogels were autoclaved at 121^o^C for 30 min and incubated in 1x PBS at 37^o^C. Buffers were collected and replaced at 1, 7, 14, and 28 days. The samples were frozen until further use. The samples were lyophilized and reconstituted in a 5x smaller volume to concentrate the samples. A drop of each sample was placed and dried on a glass slide, which was then characterized using Raman spectroscopy and FTIR spectroscopy. The remaining sample was characterized using ^1^ H NMR. Gel permeation chromatography was also performed using an Agilent Infinity 1260 equipped with a PL Aquagel-OH 30 column. The mobile phase was 0.2 M NaNO_3_ and 0.01 M NaH_2_PO_4_ eluted at a flowrate of 0. 5 mL/min. Samples were filtered through a 0.22 μm membrane prior to the run.

**Statistical Analysis.** Continuous variables are expressed as mean ± SEM (standard error). For normally distributed data sets with equal variances, a two-tailed Student’s t-test was carried out to determine significance. In all cases, significance was defined as *p* ≤ 0.05. Statistical analysis was carried out using MATLAB R2018a.

## Results and discussion

**Fig. 1:**
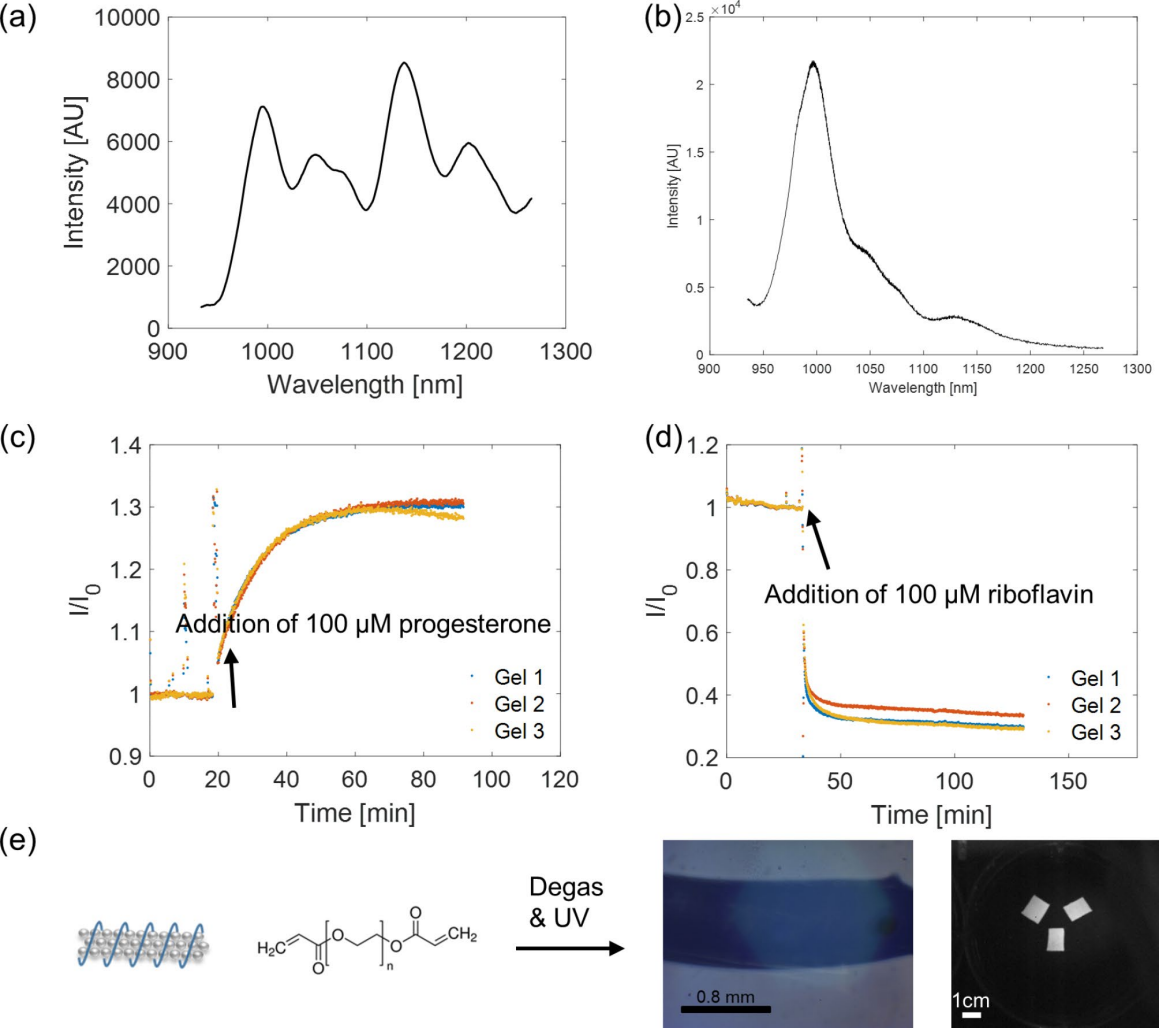
*In vitro* sensor characterization. (a) Fluorescence emission spectrum at 785 nm excitation of SM8-3-wrapped HiPCO SWNT encapsulated in PEGDA-8000 hydrogel. (b) Fluorescence emission spectrum at 785 nm excitation of ss(AAAT)_7_-wrapped (6,5) CoMoCAT SWNT encapsulated in PEGDA-8000 hydrogel. (c)SM8-3-wrapped HiPCO SWNT hydrogel fluorescence increases with an addition of 100 μm progesterone in 1X PBS solution (n = 3). (d) (AAAT)_7_-wrapped (6,5) CoMoCAT SWNT fluorescence decreases with an addition of 100 μm riboflavin in 1X PBS solution. (e) Schematics of synthesis of the PEGDA hydrogels: monomer mixed with SWNT solution and initiator, degassed, pipped between two glass slides to control for thickness, and exposed to UV light to initiate free radical polymerization

As a model sensor implant, we have fabricated p(acrylic acid_54_-*ran*-styrene_22_-ran-acrylated cortisol_4_) [28]-wrapped SWNT (SM8-3) sensors that are responsive to progesterone [28] (Fig. 1a&c) and ss(AAAT)_7_-wrapped SWNT sensors that are responsive to riboflavin [29] (Fig. 1b&d). We then encapsulated them with biocompatible PEGDA hydrogels (Fig. 1e). These sensors are enabled by Corona Phase Molecular Recognition (CoPhMoRe) [30] whereby a fluorescent nanoparticle is wrapped with an amphiphilic polymer, the hydrophilic part of which provides the dispersion colloidal stability in aqueous solutions. The adsorbed phase of the polymer on the SWNT forms a corona phase and with the nanoparticle as a whole acting as a synthetic molecular binding unit and the reporter of such events. Upon analyte binding, the nanomaterial characteristic fluorescence changes, and molecular recognition takes place when there is a selective modulation of the fluorescence toward a specific type of molecules.

Table 1 summarizes the five hydrogel formulations evaluated in this work, chosen to evaluate tissue responses when the hydrogels possess differences in SWNT concentration, SWNT wrapping, pore size, and compressive modulus. Previous *in vitro* studies have examined cellular toxicity in the presence of SWNT and have reported different results depending on the synthesis method and associated impurities in the raw SWNT material [11], SWNT corona [12, 13], and cell type [13, 14]. For example, while SDS and SDBS-wrapped HiPCO SWNT were shown to be toxic to 132N1 human astrocytoma cells, utilizing sodium cholate or ssDNA-wrapped SWNT eliminated such adverse reactions [13]. The disparate results among the same cell type indicate that each SWNT suspension may need to be tested individually to determine cellular toxicity. Furthermore, A549 and NHBE cells, both primary human lung epithelial cells, exhibited different toxic responses when exposed to dipalmitoylphosphatidylcholine-wrapped HiPCO-SWNT, indicating that particle toxicity is not universal among different cell types [16, 31]. The wide range of cellular responses may indicate that the *in vivo* response to implanted SWNT may also need to be evaluated in each type of implantation site, whether that be subcutaneous, intramuscular, etc.

**Fig. 2:**
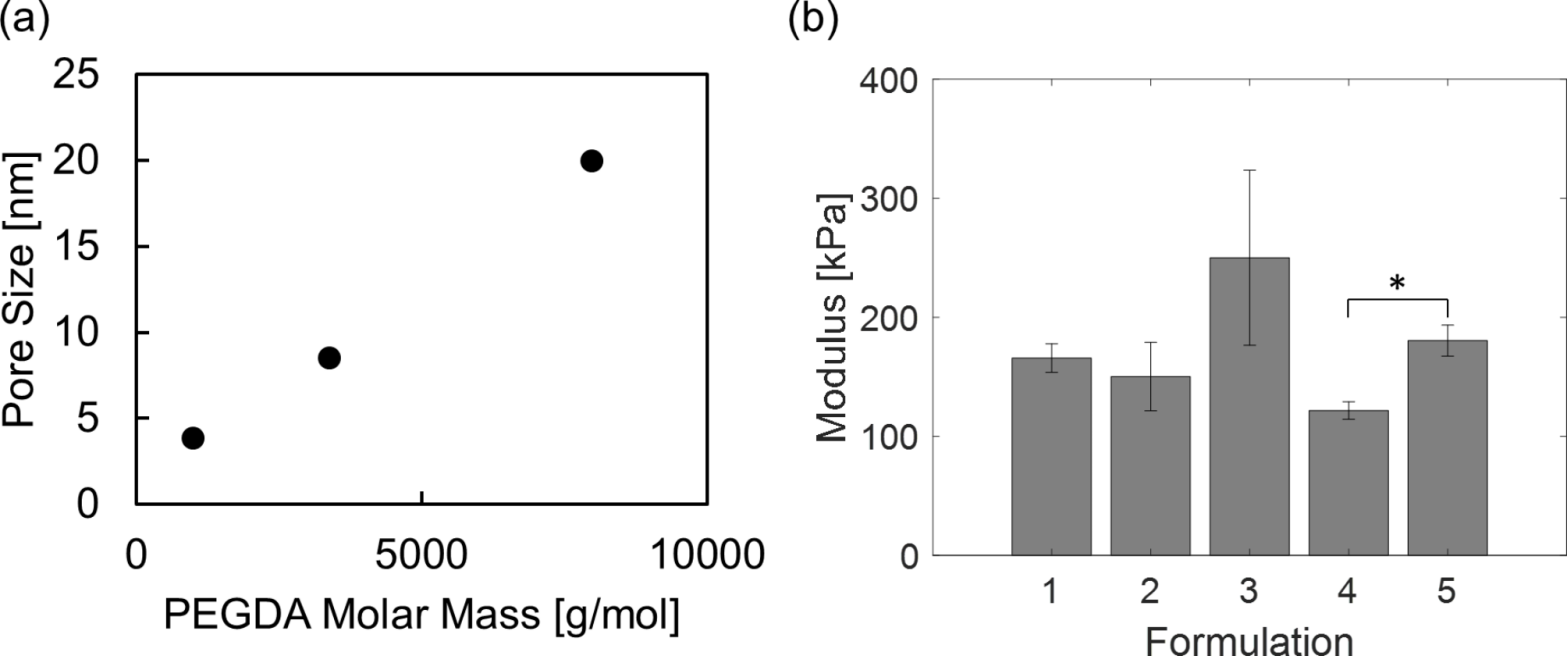
(a) Hydrogel pore sizes obtained via swelling experiments. (b) Compressive moduli (data presented as mean ± SEM, n = 2 for Formulation 3, and n = 3 for the rest, P-values are calculated using two-tailed Students’ t-test, *P < 0.05) of hydrogels obtained from the linear regions of dynamic mechanical analysis (Figure S1)

The interaction of hydrogel materials with tissue has been a widely investigated topic in literature, and we point readers to the excellent reviews written on this subject [32–34]. Conversely, there have been relatively few studies that have examined the use of hydrogels as an implantation vehicle for nanosensors, especially SWNT based nanosensors, and the effects of formulation on overall functionality. We examined the use of two different molecular weight PEGDA hydrogels. As shown in Fig. 2, the unloaded PEGDA 1000 hydrogels have a modulus of 180 kPa and a pore size of 4 nm, while the unloaded PEGDA 8000 hydrogels have a modulus of 120 kPa and a pore size of 20 nm. In addition, statistical analysis has indicated that the presence of SWNT does not have a significant impact on the hydrogel modulus. It has been demonstrated that stiffer PEGDA hydrogels elicit a more severe in vivo foreign body response [35]. However, with encapsulated nanosensors, stiffer hydrogels that have smaller pore sizes may increase the efficiency of nanoparticle encapsulation and consequently decrease the rate of product release upon degradation of the hydrogel scaffold.

**Fig. 3:**
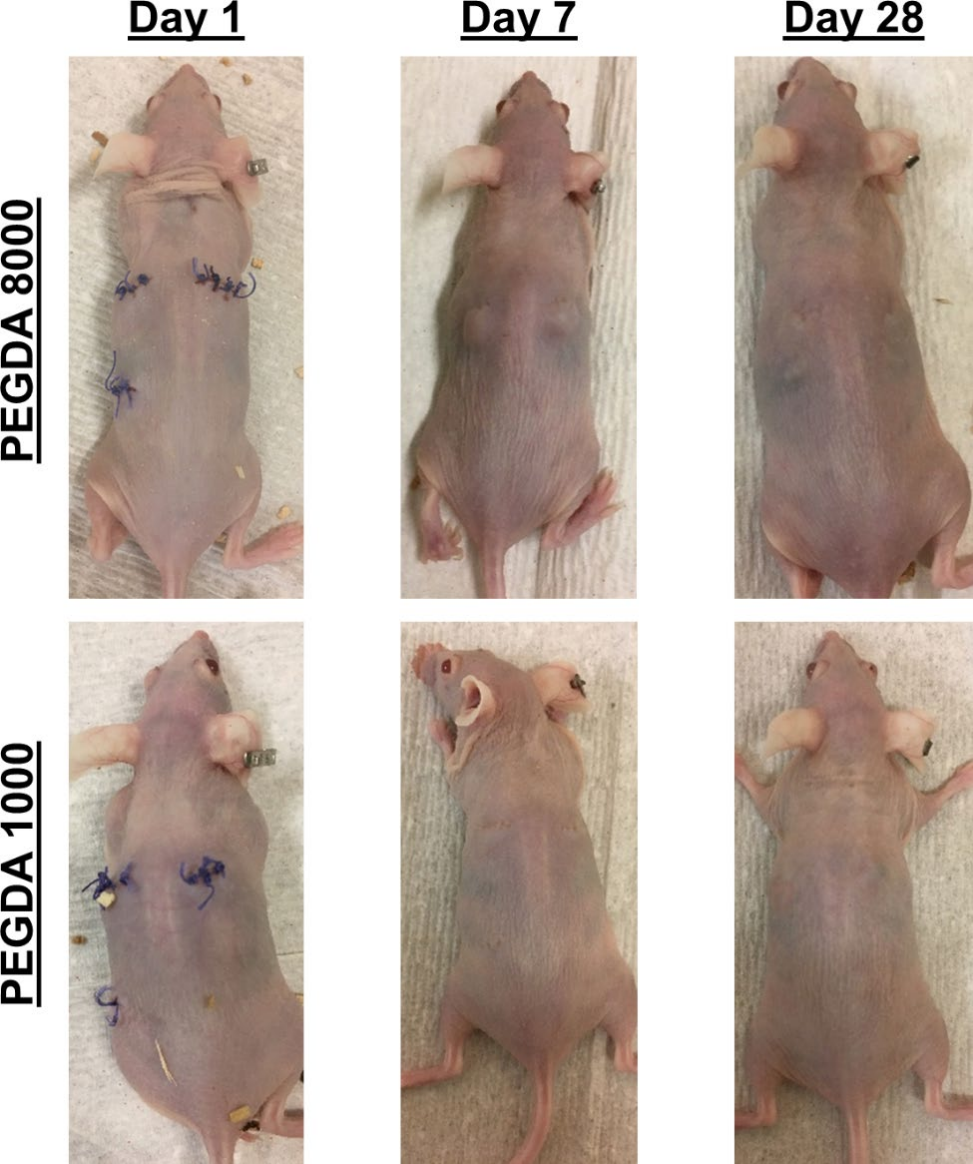
Images of mice implanted with PEGDA 8000 and PEGDA 1000 hydrogels. Mice with PEGDA 8000 hydrogels showed observable swelling in the vicinity of the implants on day 7, whereas none were noticed with the PEGDA 1000 hydrogels

The hydrogel encapsulated nanosensors were subcutaneously implanted into SKH-1E mice (Fig. 3), and tissue responses to the five hydrogel formulations were evaluated and scored according to several criteria, including the inflammatory cellular infiltrate at and around the implant site, fibrosis, edema, neovascularization, and the presence of multi-nucleated giant cells (MNGCs) [36]. The identity of the cells surrounding the implant indicates tissue tolerance of the implant, as well as the progression of healing [37]. In the classical wound healing process, the first 3–4 days are characterized by acute inflammation, in which the primary cell types are neutrophils and mast cells, which attempt to phagocytose the material. They also release degranulation molecules for the degradation of foreign material and cytokines for the progression of later stages of inflammation. Following acute inflammation, neutrophils are replaced with macrophages, which release reactive oxygen species (ROS) and attempt to phagocytose any foreign material. If the material is too large, as is the case with many implants, macrophages fuse and form giant cells. Furthermore, a fibrous capsule forms around an implant if it is not degraded. In the case of poorly biocompatible materials, deviations from the classical wound healing response may result, which may manifest itself in the presence of different cell types at delayed or accelerated timelines [38]. Furthermore, the ultimate thickness of the fibrous capsule surrounding an implant also directly indicates how well the tissue tolerates the implant [25]. Taken together over time, inflammatory infiltrate and fibrous capsule thickness provide several criteria to quantify local tissue response to implants.

**Fig. 4:**
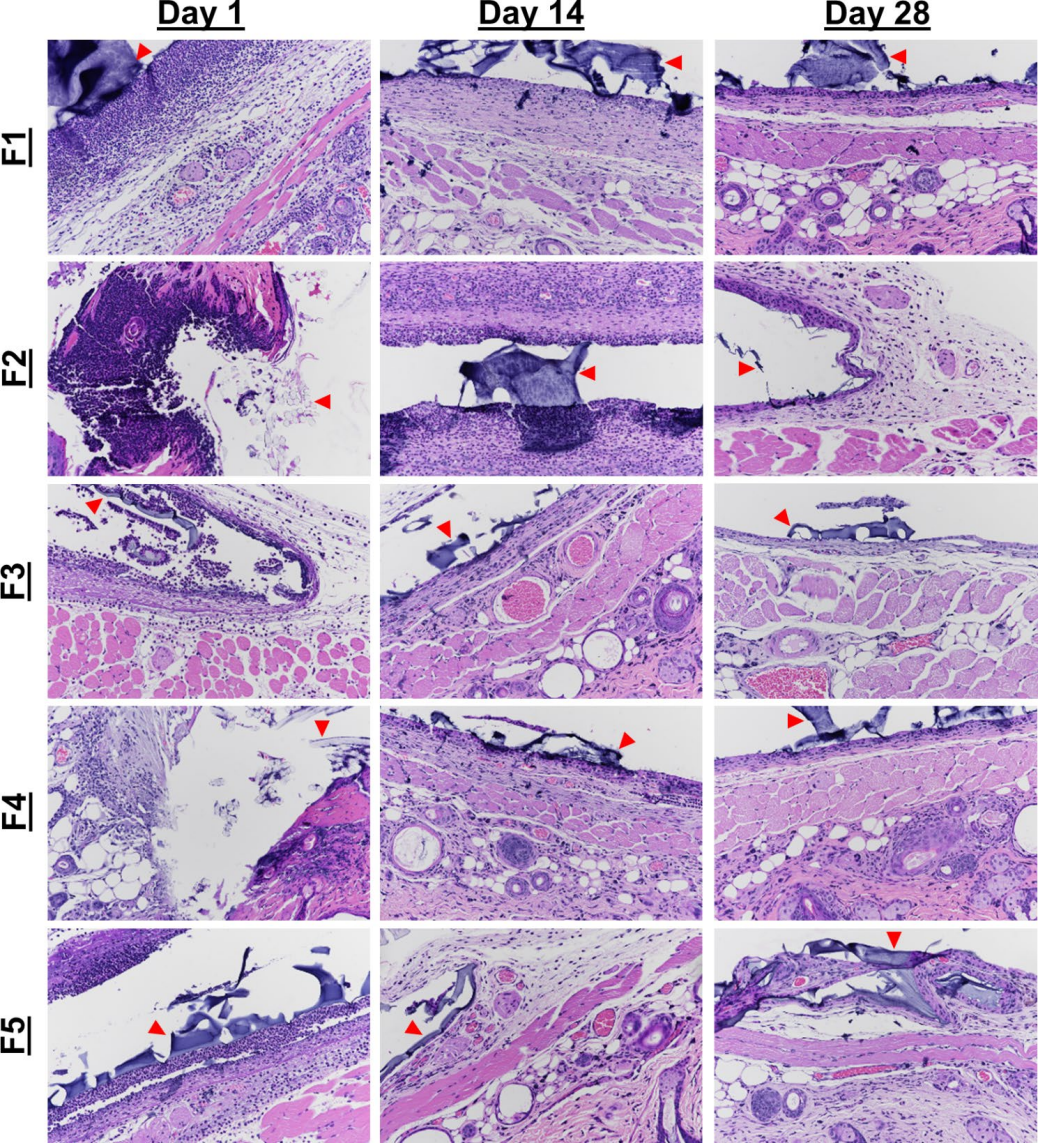
H&E stained tissue samples of SKH1-E taken from implant sites of hydrogels. Hydrogels of formulations 1–5 were explanted at days 1, 7, 14, and 28. The hydrogel itself or locations of the hydrogels are marked by arrows. In all formulations, we see heavy neutrophilic infiltration on day 1, with less on day 14, and resolution by day 28. The severity of acute inflammation is higher in formulations 1 and 2 compared to 4 and formulation 3 relative to formulation 5. Formulation 3, however, has fewer neutrophils than formulations 1 and 2. All formulations show an increase in edema, neovascularization, and fibrosis with time. Images were taken at 20x magnification, and gels are indicated by red arrows

**Fig. 5:**
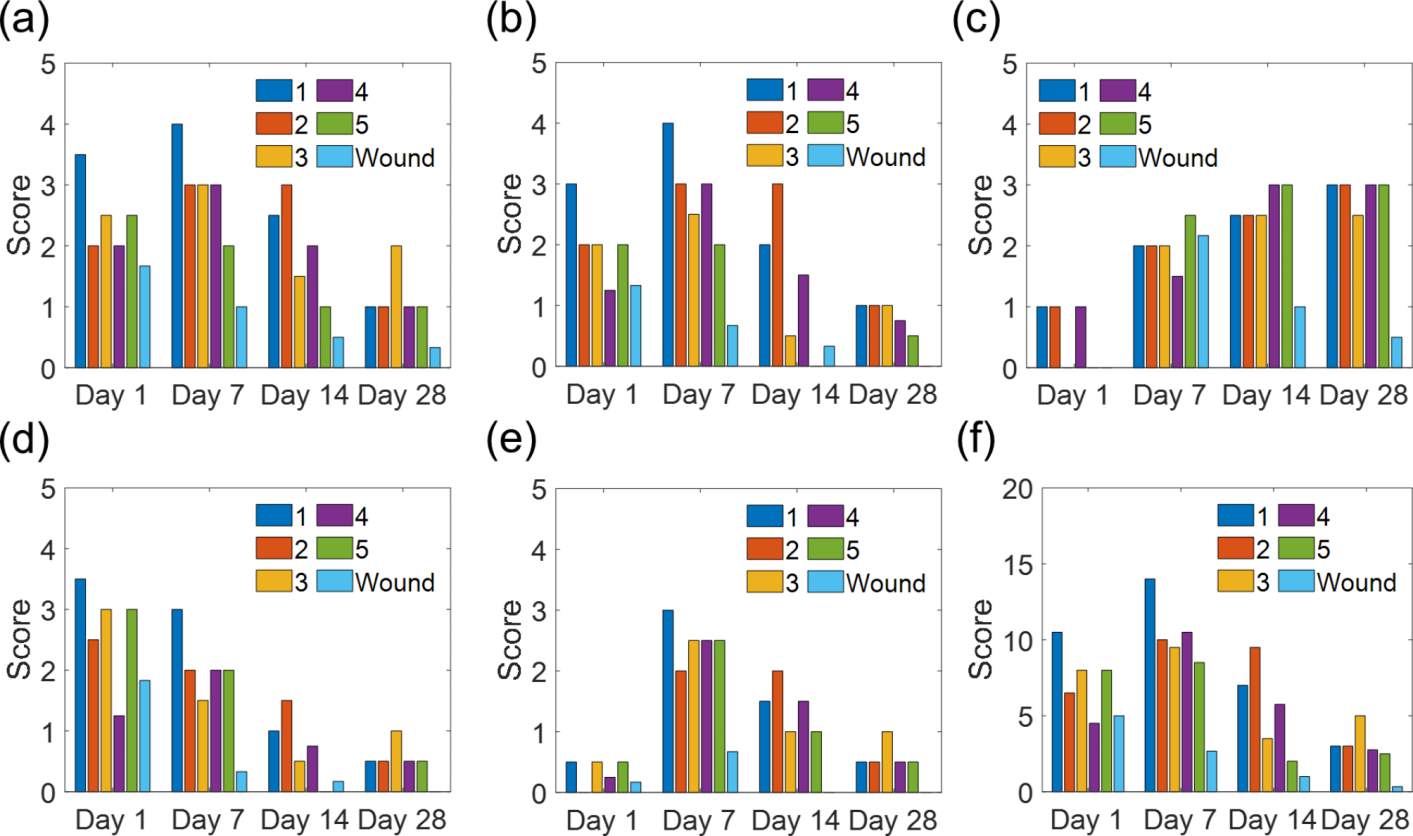
Tissue response scores for the (a) implant site, (b) tissue surrounding the implant, (c) fibrosis, (d) edema, (e) neovascularization, and (f) total adverse tissue reaction. The inflammation at and surrounding the implant site, edema, and neovascularization were rated on a scale of 0 to 4: 0 is absent, 1 is minimal, 2 is mild, 3 is moderate, and 4 is severe. Fibrosis was rated on a scale of 1 to 3, with 1 being only a mild fibrous encirclement, 2 being moderate or poorly organized fibrous encirclement, and 3 being a well-organized and epithelioid histiocytic cap. The total adverse tissue reaction was obtained by summing all the components except fibrosis. (data presented as mean, n = 3 for formulation 3, and n = 2 for the other formulations)

H&E-stained tissues are shown in Fig. 4, and the inflammation scores are summarized in Fig. 5. Among all five formulations, we observed a sequence of cellular morphologies consistent with the classical wound healing responses, with neutrophils early and macrophages later (Fig. 5a-b). As expected, fibrosis generally increased over time, as the fibrotic capsule became more organized and better defined (Fig. 5c), while edema and acute inflammation decreased with time (Fig. 5a-b,d). Neovascularization showed a maximum at day 7, with a gradual decrease in all formulations with time (Fig. 5e). Neutrophils were most numerous around the implant sites at days 1 and 7 for all formulations. By day 28, acute inflammation, fibrosis, edema, and neovascularization were of similar levels in all formulations.

Neutrophilic density around the implant revealed a significant trend: more densely cross-linked hydrogels led to a faster resolution of acute inflammation, as can be seen by comparing the scores of formulations 1&3 and 4&5 at day 14. This was also observed by the external appearance of the wounds, with obvious tissue response persisting at day 7 in PEG-8000 hydrogel that was absent in PEGDA-1000 hydrogels (Fig. 3). These differences may be explained by the differences in crosslink density of the hydrogels, with smaller pore-sized hydrogels encapsulating SWNT more efficiently and releasing fewer degradation products. By day 28, however, all five hydrogel formulations showed a similar amount of inflammation at the site. Another important observation is that at the early time points, formulation 1 had the highest amount of acute inflammation, possibly a consequence of the wrapping. Formulation 1 contained poly (acrylic acid-*ran*-styrene-*ran*-acrylated cortisol)-wrapped SWNT, while formulation 2 contained ss(AAAT)_7_-wrapped SWNT, indicating that the corona also influences the inflammatory response, possibly due to imperfect encapsulation of the SWNT and/or release of loosely bound wrapping molecules from the gel. It is also possible that proteins naturally present in the body diffuse into the hydrogel and get denatured. The denatured proteins then diffuse out of the hydrogel and trigger foreign body responses.

The degradation products were monitored by taking aliquots of buffer in which hydrogels were incubated at 37^o^C. Possible products include individually wrapped SWNT, SWNT aggregates, free wrapping polymer, and degradation products of the hydrogel matrix and/or wrapping polymer. Aliquots were taken at 1, 7, and 12 days. Gel permeation chromatography (GPC) was used to detect any polymeric degradation products. Degradation products were not concentrated enough to be detected by GPC, even after the concentration of the products with lyophilization and reconstitution step. Furthermore, SWNTs were also not detected in the incubation buffer using Raman spectroscopy. The analysis of degradation products suggests that SWNT and/or wrapping molecule leakage is not the main cause of the high acute inflammation in formulation 1.

**Fig. 6:**
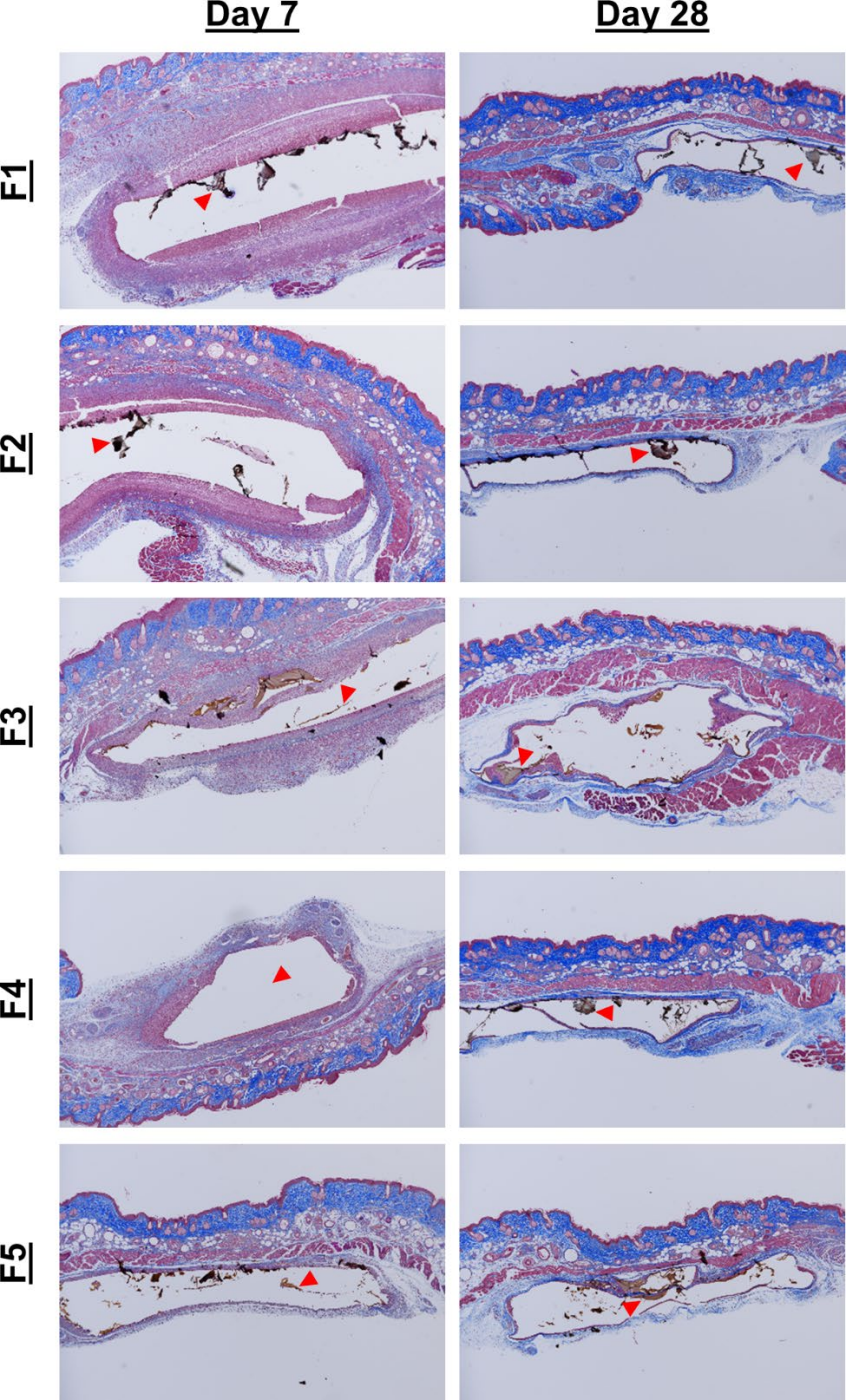
Masson’s Trichrome stained tissue samples imaged at 4x magnification. The progression of healing can be seen by observing the regions of blue, representing fibrous tissue, increasing from day 7 to day 28. Formulations 3–5 appear to have slightly more organized fibrous regions at day 7 compared to formulations 1 and 2, indicating faster healing with smaller pore sizes, as well as lower SWNT concentrations. The hydrogel itself or locations of the hydrogels are marked by arrows

Masson’s trichrome staining was performed to visualize the fibrous capsule formed around the implants (Fig. 6). At day 7, we observed the beginning of fibrous capsular formation in the implant and surrounding the implant, with more organized fibrosis in formulations 3, 4, and 5. These indicate that healing is occurring more quickly in hydrogels without SWNT, as well as in hydrogels with SWNT having smaller pore sizes. By day 28, we see that the fibrous capsules have fully encircled the implant in all formulations. These results are consistent with the H&E staining, which was expected given the role macrophages and fibroblasts play in capsule formation.

**Fig. 7:**
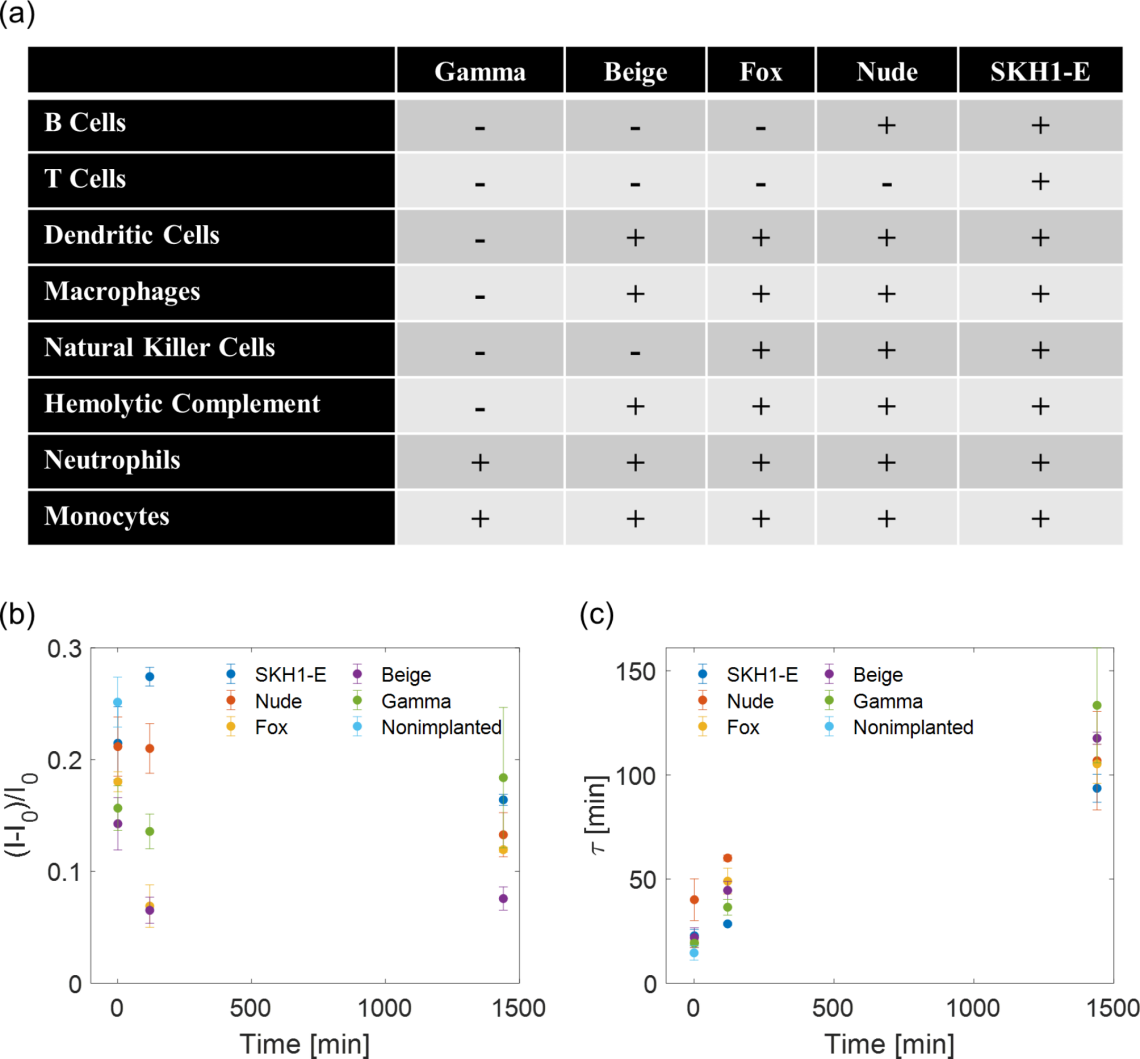
(a) Summary of the functional and dysfunctional inflammatory cells in five mice lines used in this study. Hydrogel sensors were implanted for a time period (1 min, 2 h, 24 h), explanted, and tested outside the mice against 100 µM progesterone. (b) The maximum sensitivity decreased with increasing implantation time in general. (c) For all mice lines, the kinetics of response slowed with longer implantation time

Inflammatory cells release reactive molecules that serve to deactivate pathogens and digest residual materials [39]. To determine if this inflammatory response interferes with the function of a SWNT sensor implant irreversibly, formulation 1 hydrogels were implanted in five lines of mice with varying functional inflammatory cells (Fig. 7a). The hydrogels were explanted at a given time between 0 and 24 h, incubated in 1x PBS to remove any reversibly bound analytes, and challenged with 100 µM progesterone. The responses were evaluated in terms of the maximum magnitude of sensor response and the time constant to reach 66% of the maximum response. The results for the mice are summarized in (7b-c). In general, the maximum sensitivity of the hydrogels to progesterone decreased upon implantation and the kinetics of responsivity slowed with longer implantation times. Given the time scale of the implantation (~ 24 h), acute inflammation would be the active tissue response because of the surgical procedure, even in the case of a completely inert implant. Though the mice lines had unique populations of functional immune cells, there was no clear trend in the extent to which the sensors were deactivated or slowed. It is important to note, however, that they all had functional neutrophils and monocytes, which would be active at this time scale [37]. Neutrophils release degranulation molecules which have the potential to chemically alter the SWNT corona and thus the recognition capability of the nanoparticle [39]. Furthermore, protein fragment adhesion might occur immediately upon implantation, potentially clogging the porous hydrogel, and effectively increasing the time required for analyte diffusion into the hydrogel [25]. This clog may have further consequences on the sensitivity as well, in that individual SWNT are entrapped at specific locations in the hydrogel, which may end up inaccessible to the analyte and effectively be trapped in an unresponsive state. Altogether, the time dependence of these data, despite working in serum and an incubation period in the buffer to remove reversibly bound interfering molecules, suggests that the progression of inflammation may in fact disrupt sensor functionality.

## Conclusions and future work

In this work, tissue responses were tracked in response to nanosensors encapsulated in a hydrogel matrix, using SWNT based biosensor as an example. Though inflammations are resolved in all five formulations at day 28, the resolution rate of acute inflammation is highly dependent on SWNT wrappings, with DNA wrappings leading to less severe acute inflammation. Furthermore, comparisons between formulations 1 and 3 indicate that changing the physical parameters of the hydrogel such as cross-link density may reduce the overall nanoparticle release, leading to a better-tolerated implant with faster resolution of inflammation. The deactivation of SWNT sensor functionality during the acute inflammatory response was demonstrated. Altogether, these results suggest design considerations when formulating hydrogel-encapsulated nanosensors. Future work will examine further hydrogel formulations beyond PEGDA hydrogels to observe the chemical dependence of the scaffolding material on the tissue response. With a larger collection of hydrogel formulation data, an optimal hydrogel may be chosen that best extends the longevity of nanosensors *in vivo.*

## Electronic supplementary material

Below is the link to the electronic supplementary material.

Supplementary Material 1

## Data Availability

The raw/processed data required to reproduce these findings cannot be shared at this time as the data also forms part of an ongoing study.

## References

[1] Li Z, de Barros ALB, Soares DCF, Moss SN, Alisaraie L (2017). Functionalized single-walled carbon nanotubes: cellular uptake, biodistribution and applications in drug delivery. Int J Pharm.

[2] Oh SH et al. Nanophotonic biosensors harnessing van der Waals materials. *Nat. Commun* 2021 121 12, 1–18 (2021). 10.1038/s41467-021-23564-4 PMC8219843 34158483

[3] Rabbani M, Hoque ME, Mahbub Z, Bin. Nanosensors in biomedical and environmental applications: perspectives and prospects. Nanofabrication Smart Nanosensor Appl. 2020;163–86. 10.1016/B978-0-12-820702-4.00007-6.

[4] Koman VB et al. A wavelength-induced frequency filtering method for fluorescent nanosensors in vivo.Nat. Nanotechnol.17, (2022). 10.1038/s41565-022-01136-x 35637357

[5] Barbosa AI, Rebelo R, Reis RL, Bhattacharya M, Correlo V (2021). M. Current nanotechnology advances in diagnostic biosensors. Med Devices Sensors.

[6] Naresh V, Lee NA (2021). Review on biosensors and recent development of Nanostructured Materials-Enabled biosensors. Sens 2021.

[7] Galassi TV et al. An optical nanoreporter of endolysosomal lipid accumulation reveals enduring effects of diet on hepatic macrophages in vivo.Sci. Transl. Med.10, (2018). 10.1126/scitranslmed.aar2680 PMC6543545 30282694

[8] Williams RM (2018). Noninvasive ovarian cancer biomarker detection via an optical nanosensor implant. Sci Adv.

[9] Iverson NM (2013). In vivo biosensing via tissue-localizable near-infrared-fluorescent single-walled carbon nanotubes. Nat Nanotechnol.

[10] Lee MA (2019). Implanted Nanosensors in Marine organisms for physiological biologging: design, feasibility, and Species Variability. ACS Sens.

[11] Chowdhury I, Duch MC, Gits CC, Hersam MC, Walker SL (2012). Impact of synthesis methods on the transport of single walled Carbon Nanotubes in the aquatic environment. Environ Sci Technol.

[12] Heister E (2012). Drug loading, dispersion stability, and therapeutic efficacy in targeted drug delivery with carbon nanotubes. Carbon N Y.

[13] Dong L, Joseph KL, Witkowski CM, Craig MM (2008). Cytotoxicity of single-walled carbon nanotubes suspended in various surfactants. Nanotechnology.

[14] Jin H, Heller DA, Strano MS (2008). Single-particle Tracking of endocytosis and exocytosis of single-walled Carbon Nanotubes in NIH-3T3 cells. Nano Lett.

[15] Kumar V, Sharma N, Maitra SS (2017). In vitro and in vivo toxicity assessment of nanoparticles. Int Nano Lett.

[16] Gao Z, Varela JA, Groc L, Lounis B, Cognet L (2016). Toward the suppression of cellular toxicity from single-walled carbon nanotubes. Biomater Sci.

[17] Chiang IW (2001). Purification and characterization of single-wall Carbon Nanotubes (SWNTs) obtained from the gas-phase decomposition of CO (HiPco process). J Phys Chem B.

[18] Monzon A, Lolli G, Cosma S, Mohamed SB, Resasco DE (2008). Kinetic modeling of the SWNT Growth by CO Disproportionation on CoMo catalysts. J Nanosci Nanotechnol.

[19] Kolosnjaj-Tabi J (2010). In vivo behavior of large doses of ultrashort and full-length single-walled carbon nanotubes after oral and intraperitoneal administration to swiss mice. ACS Nano.

[20] Shrivastava R (1992). Comparison of in vivo acute lethal potency and in vitro cytotoxicity of 48 chemicals. Cell Biol Toxicol.

[21] Almeida JPM, Chen AL, Foster A, Drezek R (2011). In vivo biodistribution of nanoparticles. Nanomedicine.

[22] Jena PV (2015). A Carbon Nanotube Optical reporter maps endolysosomal lipid Accumulation and Heterogeneity. Submitted.

[23] Deloid G (2014). Estimating the effective density of engineered nanomaterials for in vitro dosimetry. Nat Commun.

[24] Harvey JD et al. Carbon Nanotube Reporter of MicroRNA Hybridization Events in Vivo. in *Nat* (Biomed. Eng 1 (4, 2017). doi:10.1038/s41551-017-0041 10.1038/s41551-017-0041 PMC5568023 28845337

[25] Swartzlander MD (2015). Linking the foreign body response and protein adsorption to PEG-based hydrogels using proteomics. Biomaterials.

[26] Peppas NA, Hilt JZ, Khademhosseini A, Langer R (2006). Hydrogels in biology and medicine: from molecular principles to bionanotechnology. Adv Mater.

[27] Pedersen JS, Sommer C (2005). Temperature dependence of the virial coefficients and the chi parameter in semi-dilute solutions of PEG. Prog Colloid Polym Sci.

[28] Lee MA (2020). Implantable nanosensors for human steroid hormone sensing in vivo using a self-templating Corona Phase Molecular Recognition. Adv Healthc Mater.

[29] Bakh NA (2021). Transcutaneous Measurement of essential vitamins using Near-Infrared fluorescent single-walled Carbon Nanotube Sensors. Small.

[30] Zhang J (2013). Molecular recognition using corona phase complexes made of synthetic polymers adsorbed on carbon nanotubes. Nat Nanotechnol.

[31] Herzog E (2009). SWCNT suppress inflammatory mediator responses in human lung epithelium in vitro. Toxicol Appl Pharmacol.

[32] Caló E, Khutoryanskiy VV (2015). Biomedical applications of hydrogels: a review of patents and commercial products. Eur Polym J.

[33] Saboktakin M, Tabatabaei RM (2015). Supramolecular hydrogels as drug delivery systems. Int J Biol Macromol.

[34] Dimatteo R, Darling NJ, Segura T (2018). In situ forming injectable hydrogels for drug delivery and wound repair. Adv Drug Deliv Rev.

[35] Blakney AK, Swartzlander MD, Bryant SJ (2012). The effects of substrate stiffness on the in vitro activation of macrophages and in vivo host response to poly(ethylene glycol)-based hydrogels. J Biomed Mater Res Part A.

[36] van Rijssel EJC, Trimbos JB, da Costa A, Fleuren GJ, Brand R (1988). Assessment of tissue reaction at suture knots; an adaptation of Sewell’s scoring system. Eur J Obstet Gynecol Reprod Biol.

[37] Klopfleisch R, Jung F (2017). The pathology of the foreign body reaction against biomaterials. J Biomed Mater Res Part A.

[38] Zhu Z (2017). An overview of Carbon Nanotubes and Graphene for Biosensing Applications. Nano-Micro Lett.

[39] Selders GS, Fetz AE, Radic MZ, Bowlin GL (2017). An overview of the role of neutrophils in innate immunity, inflammation and host-biomaterial integration. Regen Biomater.

