# Supplementary material for: In-Vivo fluorescent nanosensor implants based on hydrogel-encapsulation: investigating the inflammation and the foreign-body response
Source: J Nanobiotechnology. 2023 Apr 24;21:133. doi: 10.1186/s12951-023-01873-8 (PMC10123989; doi:10.1186/s12951-023-01873-8)
Supplement: Supplementary file 1 — Supplementary Material 1 [file 12951_2023_1873_MOESM1_ESM.docx]

Supporting Information for

***In-Vivo* Fluorescent Nanosensor Implants Based on Hydrogel-Encapsulation: Investigating the Inflammation and the Foreign-Body Response**

Michael A. Lee,^1§^ Xiaojia Jin, ^1§^ Sureshkumar Muthupalani,^2^ Naveed A. Bakh, ^1^ Xun Gong,^1^ Michael S. Strano^1*^

1. Department of Chemical Engineering, Massachusetts Institute of Technology, Cambridge, Massachusetts 02139, USA

2. Division of Comparative Medicine, Massachusetts Institute of Technology, Cambridge, Massachusetts 02139, USA

§ These authors contributed equally to this work

*Corresponding author: [strano@mit.edu](mailto:strano@mit.edu)

Table of Contents:

**Figure S1**. True stress-strain plots of the different hydrogel formulations


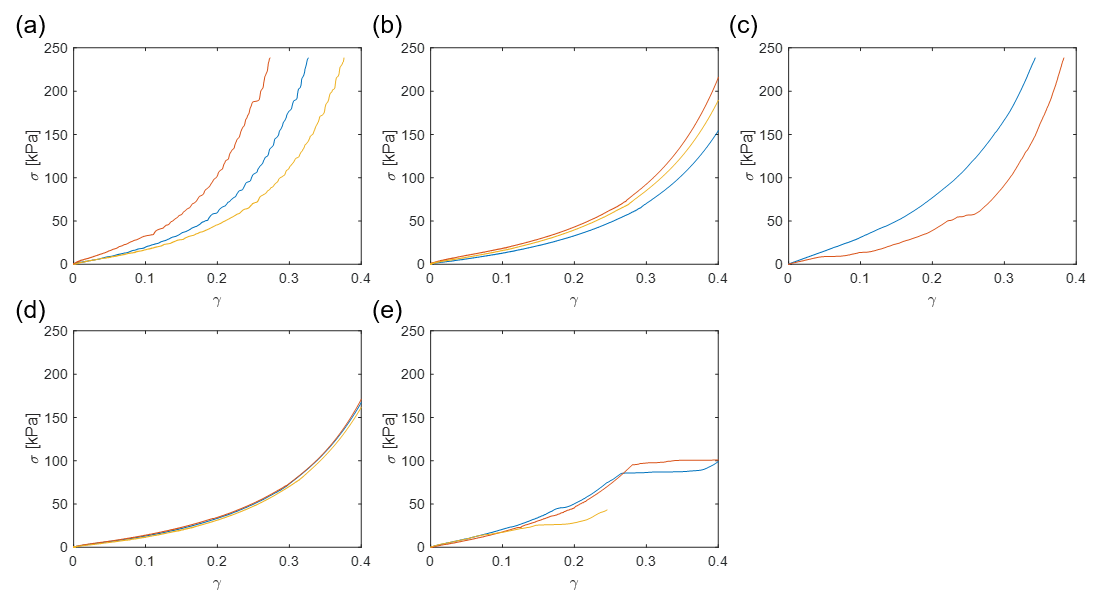


**Figure S1**. True stress-strain plots of the different hydrogel formulations, where the linear region of the plot was used to calculate the compressive moduli. (a) Formulation 1; (b) Formulation 2; (c) Formulation 3; (d) Formulation 4; (e) Formulation 5.
